# 广泛期小细胞肺癌免疫治疗：研究进展和未来展望

**DOI:** 10.3779/j.issn.1009-3419.2024.102.35

**Published:** 2024-11-20

**Authors:** Yuling ZHONG, Jingyi WANG, Lin WU

**Affiliations:** ^1^421001 衡阳，南华大学湖南省肿瘤医院研究生协作培养基地（钟雨玲）; ^1^Graduate Collaborative Training Base of Hunan Cancer Hospital, University of South China, Hengyang Medical School, Hengyang 421001, China; ^2^410013 长沙，湖南省肿瘤医院/中南大学湘雅医学院附属肿瘤医院胸内二科（钟雨玲，王婧怡，邬麟）; ^2^The Second Department of Thoracic Oncology, Hunan Cancer Hospital/The Affiliated Cancer Hospital of Xiangya School of Medicine, Central South University, Changsha 410013, China

**Keywords:** 肺肿瘤, 免疫治疗, 免疫检查点抑制剂, 生物标志物, Lung neoplasms, Immunotherapy, Immune checkpoint inhibitors, Biomarkers

## Abstract

目前，免疫联合化疗已经成为广泛期小细胞肺癌（extensive-stage small cell lung cancer, ES-SCLC）的一线治疗标准方案。近年来，免疫检查点抑制剂（immune checkpoint inhibitors, ICIs）在肺癌领域受到了广泛的关注和研究。同时，在此过程中也伴随着诸多挑战与考验，如生物标志物的探索、新靶点新模式的探讨和特殊人群管理等。本文综述了ES-SCLC免疫治疗领域的研究进展，并据此展望该领域的未来发展趋势与潜在方向。

## Abstract (en)

At present, immunotherapy combined with chemotherapy has become the first-line standard of treatment for extensive-stage small cell lung cancer (ES-SCLC). In recent years, immune checkpoint inhibitors (ICIs) have received extensive attention and research in the field of lung cancer. At the same time, there are many challenges and tests in this process, such as the exploration of biomarkers, the exploration of new targets and new models, and the management of special populations. This article reviews the research progress in the field of ES-SCLC immunotherapy, and looks forward to the future development trend and potential direction of this field.

《2024年癌症统计报告》数据^[1]^显示，肺癌是中国乃至全球发病率和死亡率最高的恶性肿瘤，其中小细胞肺癌（small cell lung cancer, SCLC）占肺癌组织类型的15%。SCLC恶性程度高、异质性大、侵袭性强，极易发生远处转移^[2]^，患者确诊时大多数为晚期，预后极差。SCLC对放化疗极为敏感，但患者的长期生存有限^[3]^，改善患者预后是亟需解决的临床问题。近年来，免疫检查点抑制剂（immune checkpoint inhibitors, ICIs）已被广泛批准用于肺癌治疗，免疫治疗联合含铂双药化疗在近20年来首次被证实可以改善广泛期SCLC（extensive-stage SCLC, ES-SCLC）的预后^[4]^。然而，尽管取得了诸多进展，但SCLC的免疫治疗仍面临着诸多挑战，如生物标志物的探索和特殊人群管理等。本文旨在综述并总结关于ES-SCLC免疫治疗领域的研究进展，分析当前ES-SCLC免疫治疗所面临的多重挑战，并据此展望该领域的未来发展趋势与潜在方向。

## 1 免疫检查点作用机制

目前，常见的ICIs主要包括以下几种类型：程序性细胞死亡受体1（programmed cell death 1, PD-1）抑制剂、程序性细胞死亡配体1（programmed cell death ligand 1, PD-L1）抑制剂、细胞毒性T淋巴细胞相关抗原4（cytotoxic T lymphocyte-associated antigen 4, CTLA-4）和delta样配体3（delta-like ligand 3, DLL3）等。PD-1与PD-L1的结合促成肿瘤细胞免疫逃逸，触发T细胞的负向调节通路，减弱其活性。PD-1/PD-L1抑制剂通过阻断这一过程，提高T细胞对肿瘤细胞的直接攻击能力，并促进其在肿瘤微环境中的扩散和增殖，强化抗肿瘤免疫反应。CTLA-4抑制剂则主要作用于免疫反应的早期阶段，通过阻止CTLA-4与B7分子的相互作用来抑制T细胞的过度活化，并调节Treg细胞的抑制作用，以保持免疫系统的稳定。DLL3抑制剂^[5]^可阻断配体DLL3与Notch受体的免疫负性调控，从而影响细胞的增殖、周期阻滞和凋亡等过程。

## 2 ES-SCLC一线免疫治疗

近年来，免疫治疗联合化疗在ES-SCLC治疗中取得关键进展（详见表1^[6⇓⇓⇓⇓⇓⇓⇓⇓⇓⇓⇓⇓⇓⇓⇓-22]^）。同时，ICIs联合抗血管生成、放疗等临床治疗新模式的探索也正在进行。

**表1:** ES-SCLC一线免疫治疗临床研究汇总

Trial	Phase	Arm	No. of patients	Primary endpoints	mOS (mon)	mPFS (mon)	ORR (%)	mDOR (mon)
IMpower-133^[6,7]^	III	Atezolizumab+chemotherapy vs Placebo+chemotherapy	201 vs 202	OS, PFS	12.3 vs 10.3; HR=0.70	5.2 vs 4.3; HR=0.77	60.2 vs 64.4	4.2 vs 3.9
CASPIAN^[8,9]^	III	Durvalumab+chemotherapy vs Chemotherapy	268 vs 269	OS	13.0 vs 10.3; HR=0.73	5.1 vs 5.4; HR=0.78	68.0 vs 58.0	5.1 vs 5.1
CAPSTONE-1^[10,11]^	II	Adebrelimab+chemotherapy vs Placebo+chemotherapy	230 vs 232	OS	15.3 vs 12.8; HR=0.72	5.8 vs 5.6; HR=0.67	70.4 vs 65.9	5.6 vs 4.6
PAVE^[12]^	II	Avelumab+chemotherapy	55	1-yr PFS	10.3	5.8	69.1	5.6
CeLEBrATE^[13]^	Ⅱ	Atezolizumab+Bevacizumab +chemotherapy	53	1-yr OS	12.7	6.2	-	-
BEAT-SC^[14]^	III	Atezolizumab+Bevacizumab +chemotherapy vs Placebo+Bevacizumab +chemotherapy	166 vs 164	PFS	13.0 vs 16.6; HR=1.22	5.7 vs 4.4; HR=0.70	-	-
ETER-701^[15]^	II	Benmelstobart+Anlotinib +chemotherapy vs Chemotherapy	246 vs 247	OS, PFS	19.3 vs 11.9; HR=0.61	6.9 vs 4.2; HR=0.32	81.3 vs 66.8	5.8 vs 3.1
KEYNOTE-604^[16]^	III	Pembrolizumab+chemotherapy vs Placebo+chemotherapy	228 vs 225	OS, PFS	10.8 vs 9.7; HR=0.80	4.5 vs 4.3; HR=0.75	70.6 vs 61.8	4.2 vs 3.7
CheckMate-45^[17]^	III	Nivolumab+Ipilimumab vs Placebo	279 vs 275	OS	9.2 vs 9.6; HR=0.92	1.7 vs 1.4; HR=0.72	-	10.2 vs 8.1
ASTRUM-005^[18,19]^	III	Serplulimab+chemotherapy vs Placebo+chemotherapy	389 vs 196	OS	15.8 vs 11.1; HR=0.61	5.7 vs 4.3; HR=0.48	80.2 vs 70.4	5.6 vs 3.2
RATIONALE-312^[20]^	III	Tislelizumab+chemotherapy vs Placebo+chemotherapy	227 vs 230	OS	15.5 vs 13.5; HR=0.75	4.7 vs 4.3; HR=0.64	68.0 vs 62.0	4.3 vs 3.7
EXTENTORCH^[21]^	III	Toripalimab+chemotherapy vs Placebo+chemotherapy	223 vs 219	OS, PFS	14.6 vs 13.3; HR=0.80	5.8 vs 5.6; HR=0.67	-	-
NCT04996771^[22]^	IB/II	Toripalimab+Surufatinib +chemotherapy	35	PFS	21.1	6.9	97.1	-

ES-SCLC: extensive-stage small cell lung cancer; mOS: median overall survival; mPFS: median progression-free survival; ORR: objective response rate; mDOR: median duration of response; HR: hazard ratio.

### 2.1 化疗联合PD-L1抑制剂

目前，阿替利珠单抗、度伐利尤单抗和阿得贝利单抗联合化疗方案已获美国食品药品监督管理局（Food and Drug Administration, FDA）与中国国家药品监督管理局（National Medical Products Administration, NMPA）批准，用于ES-SCLC患者的一线治疗。

IMpower-133研究^[6]^首次报道了在ES-SCLC一线免疫联合治疗中的双主要研究终点阳性结果。本研究共纳入201例接受阿替利珠单抗联合化疗的患者，研究结果显示，相较于化疗，阿替利珠单抗联合化疗组可显著提高中位无进展生存期（progression-free survival, PFS）（4.3 vs 5.2个月，P=0.01，HR=0.77）并延长中位总生存期（overall survival, OS）（10.3 vs 12.3个月，P=0.02，HR=0.70），不良反应以皮疹和甲状腺功能减退最为常见但耐受性良好。因此，2019年FDA正式批准了阿替利珠单抗联合化疗成为ES-SCLC一线治疗的新规范。此外，该研究^[7]^的3、4、5年OS率分别为16%、13%和12%，进一步巩固了其治疗优势。CASPIAN研究^[8]^是另一项随机、对照、开放的III期临床试验，结果显示，与单纯化疗相比，度伐利尤单抗联合化疗可以显著延长中位OS（10.3 vs 13.0个月，P=0.01，HR=0.73），两组不良反应相似且耐受。随后，2023年欧洲肺癌大会（European Lung Cancer Congress, ELCC）公布的结果^[9]^显示，度伐利尤单抗联合化疗组的中位OS和3年OS率分别为12.9个月和17.6%，提示该治疗方案能够为ES-SCLC患者带来持续的OS获益。CAPSTONE-1研究^[10]^是旨在评估国产PD-L1抑制剂阿得贝利单抗与化疗联合使用的有效性和安全性，研究发现，在ES-SCLC患者中，阿得贝利单抗联合化疗相较于单独化疗显著提高了生存率，中位OS延长了2.5个月（15.3 vs 12.8个月，P=0.01，HR=0.72），最常见的不良反应为血液毒性，总体安全性良好。有研究数据^[11]^显示相较于单独化疗，阿得贝利单抗联合化疗在1年（62.9% vs 52.0%）、2年（30.9% vs 17.8%）和3年OS率（21.1% vs 10.5%）方面均有所提高。此外，2024年ELCC首次报道了PAVE研究^[12]^结果，显示免疫治疗（阿维鲁单抗）与化疗联合用于一线治疗ES-SCLC是可行的（中位OS：10.3个月，中位PFS：5.8个月），虽然未达到1年PFS的主要研究终点。

随着免疫联合化疗方案呈现出可观的疗效获益，其结合抗血管生成的新模式则进一步拓宽了ES-SCLC的治疗选择。CeLEBrATE研究^[13]^评估了阿替利珠单抗联合贝伐珠单抗以及化疗治疗初治ES-SCLC患者的有效性和安全性，数据显示，该人群中位OS为12.7个月，1年OS率为61.8%（P=0.04），但需警惕以中性粒细胞减少症为主的潜在的治疗相关严重不良事件。更进一步的是，BEAT-SC研究^[14]^是首个以免疫联合化疗为对照组，评估阿替利珠单抗联合贝伐珠单抗以及化疗疗效的III期临床研究，该研究阳性结果为亚裔患者提供了长期生存获益的证据（中位OS：16.6 vs 13.0个月，P=0.22，HR=1.22；中位PFS：4.4 vs 5.7个月，P<0.01，HR=0.70），两组不良反应相似。相似的是，ETER-701研究^[15]^是一项探索贝莫苏拜单抗联合安罗替尼及化疗一线治疗ES-SCLC的III期临床研究，与标准化疗相比，四药组（贝莫苏拜单抗联合安罗替尼及化疗）一线治疗ES-SCLC，中位PFS显著延长（4.2 vs 6.9个月，P<0.01，HR=0.32），中位OS显著提高（11.9 vs 19.3个月，P<0.01，HR=0.61）。基于ETER-701研究的优秀数据，NMPA批准贝莫苏拜单抗联合安罗替尼及化疗用于ES-SCLC一线治疗。

### 2.2 化疗联合PD-1抑制剂

KEYNOTE-604^[16]^和CheckMate-451^[17]^等研究结果显示PD-1抑制剂能改善PFS，导致ES-SCLC领域临床研究激增。但这些研究未带来患者OS获益，国产PD-1抑制剂开始进行原创性研究。

ASTRUM-005研究^[18,19]^是一项对比斯鲁利单抗联合化疗与单纯化疗的有效性和安全性的随机双盲、国际多中心III期临床研究。结果显示：与安慰剂组相比，斯鲁利单抗组中位OS延长了4.7个月（11.1 vs 15.8个月），显著降低了39%的死亡风险（P<0.01, HR=0.61），中位PFS提高了1.4个月（4.3 vs 5.7个月），降低了52%的疾病进展风险（P<0.01, HR=0.48），且具有良好的耐受性。因此，斯鲁利单抗成为首个获批一线治疗SCLC的抗PD-1单抗。随后公布的长期生存结果^[19]^显示，3年OS率为24.6%，强有力地支持了这一点。RATIONALE-312研究^[20]^是另一项中国原创性研究，探索了替雷利珠单抗联合化疗一线治疗ES-SCLC的有效性和安全性。替雷利珠单抗联合化疗组的中位OS显著优于安慰剂组（15.5 vs 13.5个月，P<0.01，HR=0.75），PFS显著改善（4.7 vs 4.3个月，P<0.01，HR=0.64），3年OS率为25%，同时具有良好的安全性，其中大多数是血液学不良事件。EXTENTORCH研究^[21]^是首个达到双主要研究终点（PFS和OS阳性）的III期临床研究，旨在评估特瑞普利单抗联合化疗一线治疗ES-SCLC有效性和安全性。结果显示，与化疗组相比，特瑞普利单抗联合化疗组PFS（5.6 vs 5.8个月，P<0.01，HR=0.67）和OS（13.3 vs 14.6个月，P=0.30，HR=0.80）均有获益，且1年PFS率提升近4倍（18.1% vs 4.9%），1年OS率也显著提高（63.1% vs 54.9%），未见新增的不良反应。基于RATIONALE-312研究^[20]^和EXTENTORCH研究^[21]^，NMPA分别批准国产PD-1抑制剂替雷利珠单抗和特瑞普利单抗联合化疗用于ES-SCLC的一线治疗。同时，EXTENTORCH研究^[21]^的优异数据，为我国临床实践提供了高级别的循证医学证据。

与此同时，在免疫联合化疗及抗血管生成方案方面，NCT04996771研究^[22]^则进一步证实了PD-1抑制剂特瑞普利单抗的可行性。在可评估人群中，索凡替尼联合特瑞普利单抗及化疗中位OS和PFS分别可达到21.1和6.9个月，客观缓解率（objective response rate, ORR）和疾病控制率（disease control rate, DCR）分别为97.1%和100%，≥3级的不良反应以中性粒细胞下降最为常见且治疗耐受性良好。

两项荟萃分析^[23,24]^表明，与化疗相比，PD-1/PD-L1抑制剂联合化疗方案一线治疗ES-SCLC可显著改善OS和PFS，且不会增加不良反应发生的概率。但值得注意的是，一项纳入四项随机试验（分别为IMpower133、CASPIAN、KEYNOTE-604和EA5161研究）的荟萃分析^[25]^则表明PD-1/PD-L1抑制剂联合化疗两者之间并无显著差异（OS: HR=0.99; PFS: HR=1.10; ORR: RR=0.95）。随着越来越多的ICIs获得批准用于ES-SCLC的治疗，我们亦期望更多的大规模荟萃分析能够为临床医生提供更为有力的数据支持。另一方面，真实世界的回顾性研究则揭示了临床实践中的情况。如中国人群的一项回顾性研究^[26]^共纳入了225例患者，研究结果显示阿替利珠单抗在临床实际运用中取得了与IMpower133研究一致的获益。然而，另一项大型真实世界研究^[27]^指出，尽管一线免疫疗法已引入，ES-SCLC患者的生存率仍较低。该研究数据显示，在纳入的4308例患者中，相较于单纯化疗，无论是一线还是后线接受PD-L1联合化疗方案OS并无显著获益（一线：8.0 vs 8.3个月；后线：4.9 vs 5.6个月）。因此，我们期待更多大规模数据验证。

### 2.3 免疫治疗新靶点新模式

免疫联合化疗已成为国内外指南一线治疗ES-SCLC的I级推荐，免疫治疗新药、新靶点、新联合模式也备受关注，为ES-SCLC患者治疗带来更多的希望和选择。尽管伊匹木单抗（NCT00527735研究）^[28]^和Tremelimumab（NCT03043872研究）^[8]^两项研究均以失败告终，但CTLA-4抑制剂在ES-SCLC一线治疗领域的有效性和安全性仍有待进一步探讨。NCT04350463研究^[29]^旨在评估免疫新靶点——赖氨酸特异性去甲基化酶1（lysine-specific histone demethylase 1, LSD1）抑制剂CC90011在初治ES-SCLC的有效性，其中ORR可达10.3%。此外，免疫联合放化疗新模式也成为免疫治疗的重要突破口。NCT04562337研究^[30]^、LEAD研究^[31]^、MATCH研究^[32]^分别报道了阿得贝利单抗、度伐利尤单抗、阿替利珠单抗联合化疗及放疗一线治疗ES-SCLC的临床获益（中位PFS：10.1 vs 8.3 vs 6.9个月）和可耐受性，为患者提供了更多治疗方案选择。此外，KEYNOTE-B99研究^[33]^则更进一步地拓宽研究范围，该研究探索了在帕博利珠单抗联合化疗的基础上分别再联合免疫球蛋白样转录物4（immunoglobulin-like transcript 4, ILT4）单抗NK-4830、CD27拮抗剂Boserolimab、酪氨酸激酶受体抑制剂仑伐替尼的疗效和安全性，研究结果显示三种联合治疗方案均具有抗肿瘤活性（ORR：NK-4830 vs Boserolimab vs 仑伐替尼：65% vs 71% vs 74%），未见新增不良反应。由此可见，新靶点新模式可协同发挥抗肿瘤作用，加强患者临床获益并延长生存周期（中位OS：15.5 vs NR vs 15.8个月），但也需要谨慎面对潜在不良反应的叠加。

## 3 ES-SCLC后线免疫治疗

尽管复发性SCLC以化疗为主，但随着免疫联合化疗成为ES-SCLC一线标准治疗方案，ICIs在复发性SCLC治疗领域的探索也逐渐崭露头角（详见表2^[34⇓⇓⇓⇓⇓⇓⇓⇓⇓⇓⇓⇓-47]^）。

**表2:** ES-SCLC后线免疫治疗临床研究汇总

Trial	Phase	Treatment	No. of patients	Primary endpoints	mOS (mon)	mPFS (mon)	ORR (%)	mDOR (mon)
CheckMate-032^[34]^	I-II	Nivolumab	109	ORR	5.6	1.4	11.9	17.9
KEYNOTE-028/158^[35,36]^	Single-arm phase Ib-II	Pembrolizumab	83	ORR	7.7	2.0	19.3	NR
CheckMate-331^[37]^	III	Nivolumab vs Chemotherapy	284 vs 285	OS	7.5 vs 8.4	1.4 vs 3.8	13.7 vs 16.5	5.1 vs 5.1
IFCT-1603^[38]^	II	Atezolizumab vs Chemotherapy	49 vs 24	6 wk ORR	9.5 vs 8.7	1.4 vs 4.3	2.3 vs 10.0	-
DeLLphi-301^[39,40]^	II	10 mg Tarlatamab vs 100 mg Tarlatamab	88 vs 88	ORR	14.3 vs NR	4.9 vs 3.9	40.0 vs 32.0	-
LUPER^[41]^	I-II	Pembrolizumab +Lurbinectedin	28	ORR	11.1	5.3	46.4	11.4
2SMALL^[42]^	I	Atezolizumab +Lurbinectedin	15	Dose, safety	-	5.0	57.6	-
ChiCTR2200059911^[43]^	II	PM8002+chemotherapy	27	Safety, ORR	-	5.5	72.7	-
PASSION^[44]^	II	Camrelizumab+Apatinib	47	ORR	8.4	3.6	34.0	-
NCT04169672^[45]^	II	Toripalimab+Surufatinib	20	ORR	11.0	3.0	10.5	NR
NCT04055792^[46]^	II	Sintilimab+Anlotinib	42	PFS	12.7	6.1	56.8	-
NCT05000684^[47]^	I/II	Toripalimab +Tifcemalimab	38	Safety	-	-	26.3	NR

NR: not reached.

### 3.1 免疫单药

根据I/II期CheckMate-032研究^[34]^、KEYNOTE-028/158研究^[35,36]^，纳武利尤单抗和帕博利珠单抗都显示出抗肿瘤活性（ORR: 纳武利尤单抗：11.9%；帕博利珠单抗：19.3%），美国国立综合癌症网络（National Comprehensive Cancer Network, NCCN）指南曾推荐两者作为既往未接受过ICIs治疗的复发性SCLC后线治疗方案。相反，FDA因III期CheckMate-331^[37]^、CheckMate-451研究^[17]^和KEYNOTE-604研究^[16]^均未取得OS获益，撤回了两者在SCLC的适应证。IFCT-1603研究^[38]^旨在评估ES-SCLC一线化疗进展后阿替利珠单抗的疗效及安全性，但因未达到主要研究终点（6周ORR）以及未取得OS获益而失败。随着免疫联合化疗在一线标准治疗中的地位得到确立，后续的临床研究对新药新靶点进行了一系列的探索。塔拉妥单抗（Tarlatamab）是双特异性抗体T细胞连接器，能够同时靶向DLL3抗体与CD3，以实现独特的免疫治疗效果。在治疗复发性SCLC的I期^[39]^和II期^[40]^（DeLLphi-301研究）中，塔拉妥单抗展现了可观的抗肿瘤活性（ORR：10 mg组 vs 100 mg组：40% vs 32%）和可接受的以细胞因子释放综合征为主的不良反应。值得注意的是，虽然PD-1/PD-L1抑制剂在复发性SCLC后线治疗中未见长期生存获益，但新型免疫制剂塔拉妥单抗为后线治疗开辟了新的可能性，有望成为该领域的重要突破点。

### 3.2 免疫联合治疗

免疫单药在后线治疗中的获益，促使了大量临床研究对“免疫+”模式进行深刻探讨，如免疫联合化疗、免疫联合抗血管、双免联合治疗等。

芦比替丁与ICIs联合的研究^[48]^表明，其在治疗效果上优于传统铂类药物，因此芦比替定联合ICIs开展了一系列研究。芦比替丁分别联合帕博利珠单抗的LUPER研究^[41]^和阿替利珠单抗的2SMALL研究^[42]^均显示出ORR和中位PFS的获益（LUPER研究：ORR：46.4%，中位PFS：5.3个月；2SMALL研究：ORR：57.6%，中位PFS：5.0个月），常见以疲劳、贫血和恶心等为主的不良反应，期待进一步随访和更大样本量的III期临床研究的数据。2023年欧洲肿瘤内科学会（European Society of Medical Oncology, ESMO）报道了PM8002联合紫杉醇治疗复发性SCLC的II期优秀数据（ChiCTR2200059911研究）^[43]^，ORR高达72.7%，中位PFS延长至5.5个月，关于PM8002的多中心、开放、随机III期临床研究也正在进行中。

PASSION研究^[44]^首次尝试探索卡瑞丽珠单抗联合阿帕替尼二线治疗复发SCLC的有效性和安全性，ORR达34.0%，中位PFS和中位OS分别为3.6和8.4个月。NCT04169672研究^[45]^和NCT04055792研究^[46]^的阳性结果分别为特瑞普利单抗联合索凡替尼（中位OS：11.0个月）、信迪利单抗联合安罗替尼（中位OS：12.7个月）治疗SCLC提供了强有力的依据。

CheckMate-032研究^[34]^验证了纳武利尤单抗联合伊匹木单抗后线治疗SCLC患者的疗效和安全性的同时，开启了难治性ES-SCLC后线治疗的新探索。一项旨在评估Tifcemalimab联合特瑞普利单抗治疗复发难治SCLC的I/II期研究^[47]^显示，在可评估患者中，总体的ORR为26.3%，既往接受过ICIs治疗患者的ORR为8.3%，未经ICIs治疗患者的ORR为40.0%。

### 3.3 免疫治疗新靶点新模式

目前除PD-1/PD-L1、CTLA-4热门研究靶点外，对B7-H3抗体偶联药物（antibody-drug conjugate, ADC）、Trop-2 ADC、双特异性抗体等新药新靶点的研究也初见成效。在经治复发性SCLC中，NCT04434482研究^[49]^、NCT04145622研究^[50]^以及TROPiCS-03研究^[51]^分别展示了聚腺苷二磷酸核糖聚合酶（poly ADP-ribose polymerase, PARP）抑制剂Senaparib、B7-H3 ADC药物DS-7300、Trop-2 ADC抑制剂戈沙妥珠单抗的研究数据（ORR: 14.3% vs 52.4% vs 29%），这些临床试验表明了免疫新药新靶点的有效性和安全性，但需要更大规模的III期临床研究来进一步验证。

## 4 ES-SCLC免疫治疗的探索

### 4.1 免疫生物标志物的探索

SCLC普遍具有倍增时间短、生长比例高、异质性大和早期侵袭性强的显著特征。尽管目前一线标准为ICIs联合化疗，但ORR、PFS以及OS方面的获益依然未能达到理想状态。因此，寻找能指导免疫治疗效果的生物标志物成为当前研究的重点^[52]^，如对PD-L1表达和肿瘤突变负荷（tumor mutational burden, TMB）的深入探索和研究。

IMpower-133研究^[6]^在SCLC免疫治疗领域受限于样本类型，未能深入分析PD-L1表达。而CASPIAN^[8]^和ASTRUM-005研究^[18]^虽能评估PD-L1，但其与主要研究终点无显著关联。进一步地，KEYNOTE-604^[16]^与CAPSTONE-1^[10]^两项研究也表明，无论PD-L1表达水平如何，患者在接受免疫疗法后的获益并未显示出明显的差异。鉴于PD-L1在SCLC病例中的低表达特性^[53]^及其与免疫治疗效果之间关系的不确定性，PD-L1作为生物标志物的使用需要更严格的验证和讨论。

同样，作为生物标志物，TMB也存在其不足之处。TMB是新抗原负荷的直接体现，是衡量癌症免疫原性的一个重要替代参数。CheckMate-032研究^[34]^已经证实了TMB与PFS及OS之间存在显著的相关性。具体而言，高TMB组在1年PFS率方面，无论是单药治疗（21.2% vs NA vs 3.1%）还是双免疫治疗（30.0% vs 6.2% vs 8.0%），均显著优于低、中TMB组。同样，在1年OS率上，高TMB组也展现出更优的结果。在单药治疗中，高、中、低TMB组的OS率分别为35.2%、22.1%和23.4%。而在双免疫治疗中，高TMB组的OS率更是达到了62.4%，至于中、低TMB组的数据目前尚不明确。然而，值得注意的是，IMpower133研究、CASPIAN研究以及KEYNOTE-604研究的亚组分析^[6,8,16]^结果却显示，在ICIs联合标准化疗的治疗下，TMB与临床获益之间并未发现明显的相关性。鉴于不同研究间存在的这种相悖结果以及当前围绕TMB作为生物标志物所存在的诸多不确定性，TMB目前尚未能被广泛认可为具有高度预测能力的指标。因此，我们期待未来更多深入严谨的临床研究来挖掘验证TMB的潜力，为肿瘤治疗应用提供坚实科学依据。

### 4.2 特殊人群的治疗

中枢神经系统是肺癌常见的转移部位，患者预后通常较差。尤其值得注意的是，SCLC侵袭性强、恶性程度大，脑转移患者2年OS率不到2%，因此，该类人群的免疫管理需尤为慎重。多项临床研究对SCLC脑转移患者免疫治疗进行了亚组分析，但结论不一。CASPIAN^[8]^和ASTRUM-005^[18]^两项研究报道SCLC中发生脑转移的患者亚群可以通过免疫治疗获得显著的临床益处。然而，KEYNOTE-604^[16]^与IMpower133^[6]^研究的亚组分析结果显示，对于脑转移患者而言，免疫联合化疗的治疗方案并未展现出明显的生存优势。此外，鉴于CAPSTONE-1研究^[10]^中纳入的脑转移患者数量有限，尚无法准确评估具体疗效。鉴于样本数量有限、肿瘤异质性显著以及脑血管屏障的存在等多重挑战，对SCLC脑转移患者的研究面临了相当大的难度。我们期待未来能够涌现出更多新型免疫制剂以及创新的免疫联合疗法模式，以进一步推动脑转移患者研究的深入与发展。

### 4.3 SCLC分子病理分型

目前SCLC的4种分子亚型^[54]^被广泛认可，分别为SCLC-A、SCLC-N、SCLC-P、SCLC-Y，相关研究已经探索了各分子分型的不同特征。临床表现方面^[55]^，SCLC-Y亚型患者发生脑或骨转移的概率比SCLC-A亚型更低，发生肝转移的概率低于SCLC-N亚型患者；生存获益方面^[56]^，SCLC-Y亚型展现了SCLC中最佳的生存优势；治疗响应方面^[57]^，SCLC-P亚型显示出对某些化疗药物的敏感性，而SCLC-N亚型则对放疗有较好的反应。值得注意的是，相关性分析^[55]^表明，SCLC中的4个亚型标志物与其肿瘤免疫微环境密切相关，SCLC-Y亚型肿瘤中发现CTLA-4^+^ T细胞浸润较少，而在SCLC-A亚型中可见更多的免疫抑制受体，如FoxP3、PD-1和CTLA-4。此外研究还指出，SCLC-Y亚型的患者某些生物标志物的表达模式与其他亚型存在显著差异，这为精准医疗提供了潜在的靶点。未来需要在遗传水平上对SCLC分子亚型和肿瘤免疫微环境与免疫治疗疗效和预后之间的相关性进行深度探索分析，以提供更全面的SCLC免疫治疗预测信息。例如，LSD1机制研究^[58]^发现，LSD1抑制剂能够激活NOTCH信号通路，下调致癌基因表达并抑制癌细胞增殖。因此，SCLC-A亚型的NOTCH信号通路表达水平可能作为LSD1抑制剂治疗效果的潜在生物标志物。

## 5 小结与展望

免疫治疗在ES-SCLC的治疗领域已逐步构建起新的格局，然而，在此过程中也伴随着诸多挑战与考验，如寻找更有价值的生物标志物、深入探索新的“免疫+”模式、积极研发新型免疫抗肿瘤治疗药物以及有效管理特殊人群等。此外，为进一步提升免疫治疗的效果，越来越多的临床研究正致力于深入解析免疫耐药的具体作用机制，并积极探寻有效的应对策略。我们坚信，随着研究的不断深入和技术的持续进步，免疫治疗将为ES-SCLC患者带来显著的益处。
